# Increased resilience for manufacturing systems in supply networks through data-based turbulence mitigation

**DOI:** 10.1007/s11740-021-01036-4

**Published:** 2021-02-26

**Authors:** Dennis Bauer, Markus Böhm, Thomas Bauernhansl, Alexander Sauer

**Affiliations:** 1 Fraunhofer Institute for Manufacturing Engineering and Automation IPA, Nobelstrasse 12, 70569 Stuttgart, Germany; 2 Institute for Energy Efficiency in Production EEP, University of Stuttgart, Nobelstrasse 12, 70569 Stuttgart, Germany; 3 Institute of Industrial Manufacturing and Management IFF, University of Stuttgart, Nobelstrasse 12, 70569 Stuttgart, Germany

**Keywords:** Turbulence, Resilience, Production planning and control

## Abstract

In manufacturing systems, a state of high resilience is always desirable. However, internal and external complexity has great influence on these systems. An approach is to increase manufacturing robustness and responsiveness—and thus resilience—by manufacturing control. In order to execute an effective control method, it is necessary to provide sufficient information of high value in terms of data format, quality and time of availability. Nowadays, raw data is available in large quantities. An obstacle to manufacturing control is the short-term handling of events induced by customers and suppliers. These events cause different kinds of turbulence in manufacturing systems. If such turbulences could be evaluated in advance, based on data processing, they could serve as aggregated input data for a control system. This paper presents an approach how to combine turbulence evaluation and the derivation of measures into a learning system for turbulence mitigation. Integrated in manufacturing control, turbulence mitigation increases manufacturing resilience and strengthens the supply network’s resilience.

## Motivation

Manufacturing industry faces enormous challenges worldwide. Mass personalization leads to smaller lot sizes while simultaneously the number of variants is increasing [1, 2]. The market is changing from a seller’s market to a buyer’s market. To meet the growing importance of customer satisfaction, a high delivery reliability has to be achieved [1, 3]. Also, the market environment in which manufacturing companies operate is becoming increasingly volatile, uncertain, complex and ambiguous [4]. This became particularly evident during the COVID-19 pandemic: quarantine and other restrictions made deliveries unreliable. Entire supply networks were not able to maintain their logistic goals [5].

**Fig. 1:**
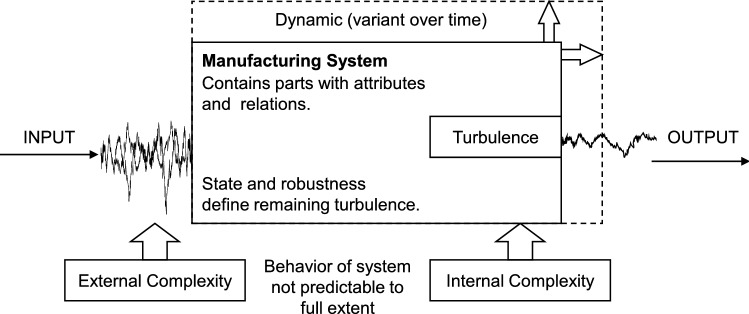
Framework of a manufacturing system in a complex environment including dampening effect of robustness

Key to operating successfully in such an environment is high resilience [6]. Manufacturing companies are contributing to their supply network’s resilience by increasing the resilience of their manufacturing system. They establish a deeper integration in their supply network and build internal mechanisms to face the complexity of their environment [1, 7]. Complexity can be understood as “the difficulty of understanding or analyzing a system” [8, 9]. Both internal and external complexity have an effect on manufacturing systems and make them in part unpredictable. Depending on the robustness of the system, the complexity results in a certain degree of remaining turbulence that needs to be mitigated. Therefore, the efficient handling of turbulence contributes decisively to the resilience of the manufacturing system. Figure 1 gives an overview of the framework.

Nowadays, regular Production Planning and Control (PPC) methods mostly use simulations and analytical approaches to find optimal schedules. They include current production load, customer order information and a model of the manufacturing system itself to simulate different scenarios and to calculate an applicable order sequence. Recent approaches also consider historical data. Seitz and Nyhuis emphasize the potential of the collection and supply and finally the utilization of operational data [10]. Gröger includes PPC methods based on operational data to form a business intelligence plattform, a data-driven optimization tool for manufacturing processes [11]. In the context of PPC, Permin et al. describe self-optimizing systems as one apporach to manage complexity. They explicitely refer to rescheduling in supply and turbulences in supply networks [12]. In general, the findings of Usuga Cadavid et al. indicate that about 75% of the observed research domains in machine-learning-based PPC, such as time estimation, smart planning and scheduling or inventory and distribution control are “barely addressed or were not explored at all” [13]. So, historical data promises to be potentially useful to further improve PPC methods. The approach of this paper is to utilize information about predicted turbulences caused by order events. In conclusion, using historical data to feed machine learning algorithms could contribute to close the missing link between existing approaches and the increasing demand for information of high value.

This leads to the following research questions: How could turbulence evaluation contribute to the information demand of PPC approaches? Moreover, how could this function be embedded in a holistic approach to control a manufacturing system by specific measures to mitigate turbulences and improve its resilience?

This paper deals with the description of the link between turbulence evaluation and the derivation of measures as well as their integration into PPC to increase the manufacturing system’s resilience. It contributes to the work in the fields of supply chain management, PPC, learning systems and autonomous manufacturing. This paper serves as a starting point to a new approach which uses feedback loops in PPC to master turbulences and thus increase the resilience of manufacturing systems in their supply networks.

## State of the art

PPC optimization in modern manufacturing is not yet completed. Therefore, this approach combines ideas and methods from different research fields to optimize PPC. Since this paper looks at manufacturing systems from the perspective of systems theory, complexity and turbulences are examined from the same point of view. Furthermore, this paper covers problems of supply networks and the principles of closed-loop control. Machine learning promises to be an essential part of the solution of this hybrid approach and therefore requires a closer look.

### Resilient systems in a complex environment

Complexity and turbulence are recurring phenomena in the manufacturing context. There are many different definitions of complexity. Brinzer et al. [14] bring forward different dimensions of complexity: Diversity, networking, dynamics and uncertainty. The evaluation of these dimensions helps to quantify complexity. Another attempt is to distinguish between internal and external complexity and to describe origins of complexity like variety, heterogeneity, dynamics and non-transparency [15]. These aspects also refere to Ashby’s concept of open systems and the requesite variety [16]. In the context of systems theory, complexity can relate to the state of a static system or its dynamic behavior [8]. Complexity can be regarded as one origin of turbulences, differentiated in subjective and objective turbulence [17]. Objective turbulences are measurable deviations from relevant indicators [18]. This means they exceed agreed tolerances. Subjective turbulences depend on the perceptions of participants in the value-adding process and occur when deviations need to be handled different from regular procedures. This paper’s understanding of turbulence is adapted from Wiendahl’s view [18]: Turbulence is the deviation of a system’s state variables mapped in the system response.

The ability to withstand influences forcing the system to respond with such deviations is called robustness [5]. The concept of robustness focuses on not changing the systems configuration and state. Systems that are capable of changing and returning to their orignal state after being altered by external influences are called *resilient*. This definition is close to the original meaning of the latin word *resilio*, which refers to the ability to rebound [19]. Holling [20] distinguishes between ecological and engineering resilience where the latter is the defintion used in this paper. It not only includes the ability to remain in a constant state [21] but to adapt under stress and, therefore, being responsive, as described by Sandler et al. [22].

A lack of robustness and resilience lead to turbulences. To detect them in data, it is suitable to examine time series of key indices. In real life, these time series describe streams of events [23]. Different approaches, like complex event processing, use time series to describe manufacturing systems in the context of systems theory [24]. The system’s state is defined by its parts, their attributes and relations. All events within the system result in recognizable patterns in data and represent a description of the system itself. Methods using this information about the black box “manufacturing system” and connecting it to PPC methods are still immature. Leading industries in automotive strive to evaluate turbulence and utilize the results for the benefit of planning and control. However, turbulences are still mainly evaluated in a qualitative approach like in interviews and workshops.

### Production planning and control in supply networks

**Fig. 2:**
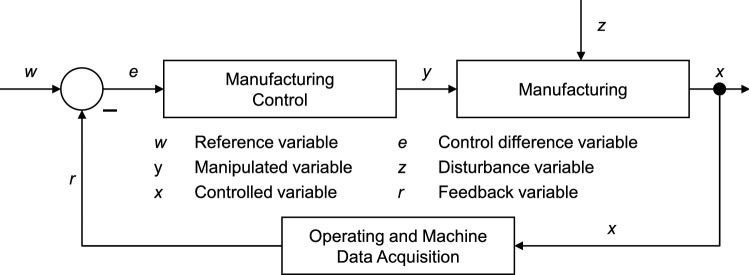
Transferring the principle of closed-loop control to production planning and control (adapted from [26])

The discussion about how to achieve logistical goals is currently dominated by the research field of production planning. However, a production plan is only useful if manufacturing control can implement the plan on the shop floor. The importance of manufacturing control is increasing, since long-term forecasts of the manufacturing state are still prone to errors. Consequently, a quick and effective reaction to turbulences is necessary. Good manufacturing control ensures robust manufacturing [3, 17, 25]. Approaches to transfer control engineering principles to PPC (cf. Fig. 2) for the creation of robust processes are receiving increasing attention [25]. Therefore, the principle of a control loop is transferred to the elements of the PPC to achieve a continuous adaptation of manufacturing.

An overview and classification of existing approaches for control loops in PPC is given in [27]. These approaches focus primarily on the internal optimization of manufacturing and not on the integration with the supply network. Furthermore, the degree of automation, especially regarding decision making, is usually lower [28]. However, due to its complexity, manufacturing can behave unexpectedly when manual interventions are made; under- or over-steering is possible if parameters are incorrectly manually adjusted [3, 29].

Supply chain event management embeds the reaction to turbulences in the context of increasing organization of manufacturing companies in supply networks [30]. Nevertheless, supply chain event management mainly focuses on the detection of events from the supply network. Reaction to these events is considered to be built on predefined rules. Therefore, it is not designed as a learning system. Adaptivity is given for known parameters only.

Since an event-driven architecture is not exclusionary to other architectural styles, it can be well implemented as a complementary approach. Therefore, supply chain event management can be combined with complex event processing to establish a more sophisticated way of how to react to events [31–35]. However, these approaches focus primarily on the design of information systems for the exchange and processing of a large number of events, not on learning systems for decisions on how to react to those events. Decisions are still mainly based on predefined rules.

### Machine learning as an enabler

Learning refers to a system which is improving its performance on future tasks after making observations about its environment [36]. In such a system, Machine Learning (ML) aims at generating knowledge from data by developing a complex heuristic model [37]. Therefore, ML becomes a key technology for the development of learning systems in the area of artificial intelligence. The automated development of models from data is where ML mainly differs from earlier approaches of artificial intelligence, which were based on manually constructed knowledge bases such as predefined rules [37, 38]. As ML allows for generalization, meaning to perform well on previously unknown data, it differs significantly from mathematical optimization [39]. Therefore, the use of ML is particularly worthwhile if, due to the complexity of the problem, not all possible situations and all changes over time can be anticipated. This is also the case, whenever it is unclear what the algorithmic solution to the problem must look like [36]. Experience has shown that ML is much more suitable than conventional methods of data analysis, especially for more than 15 dimensions in the data sets [40]. ML methods can be categorized according to the type of feedback into unsupervised learning, supervised learning and reinforcement learning [36]. While unsupervised learning does not require labeled data in the training phase, supervised learning relies on labeled data. In contrast, reinforcement learning does not require (labeled) data, but an environment that enables learning from interaction with it [36, 37]. Consequently, each of the methods is suitable for different learning tasks in manufacturing control.

The potential of methods using inductive learning increases greatly with improvements in computational technologies to process huge amounts of data. To handle classification problems, some of the best known methods are—among others—Neural Networks, Bayesian Networks, different Linear Classifiers and Decision Trees [41]. Oladipupo states that Decision Trees stand out because of high calculation speed and the possibility to improve the performance with so-called random forests. Categorizing orders into turbulence classes is a prototypical machine learning task. Models of decision forests recently gained popularity in tackling problems related to semi-automatic analysis of complex data [42].

If labeled data is not available for a sequential learning problem, e.g. scheduling in manufacturing, reinforcement learning comes into play. Reinforcement learning is suitable if a solution can be found by trial-and-error, meaning doing better can be learned by receiving feedback from an environment. Additionally, it is able to handle delayed rewards [43, 44]. As a prerequisite for the application of reinforcement learning, it is necessary to describe the problem as a Markov Decision Process (MDP). In addition, a simulation model is necessary. This is used to enable the agent to perform the trial-and-error learning safely [43, 45].

## Approach

In analogy to the Supply Chain Operations Reference Model of the Supply Chain Council, Supply Chain Management is the company’s point of entry for information on supplier- and customer-induced events [46]. These events can be either deliveries, orders or changes thereof, and therefore determine the outcome of classical production planning. The result is a planned order sequence for manufacturing, adjusted by production planning and manufacturing control. Depending on the point of time, events differ in the strength of their effects. Production planning can handle the long-term events. Short-term events, especially those related to orders that have been started, need to be handled by manufacturing control. This illustrates the sequential planning that traditionally prevails in manufacturing companies organized in job shops. A quarterly production plan is refined into a shift-granular sequence completed by dispatching decisions within minutes at each machine [47]. Feedback data from manufacturing complements this planning approach.

The availability of the relevant data is of central importance in supply networks in order to meet the need for coordination in planning and control at various levels. However, holistic and continuous information flows across all partners in a supply network are rarely available in industry. Today, data is exchanged between partners in a supply network primarily via planning objects [48]. Consequently, data that is also available in the company, for example data based on process control charts, is often used to monitor the supply network [49]. This can lead to events being detected later than would have been possible by a more in-depth exchange of data between the partners as well as a delayed forwarding to manufacturing control. The efficient response to such events therefore becomes all the more important.

**Fig. 3:**
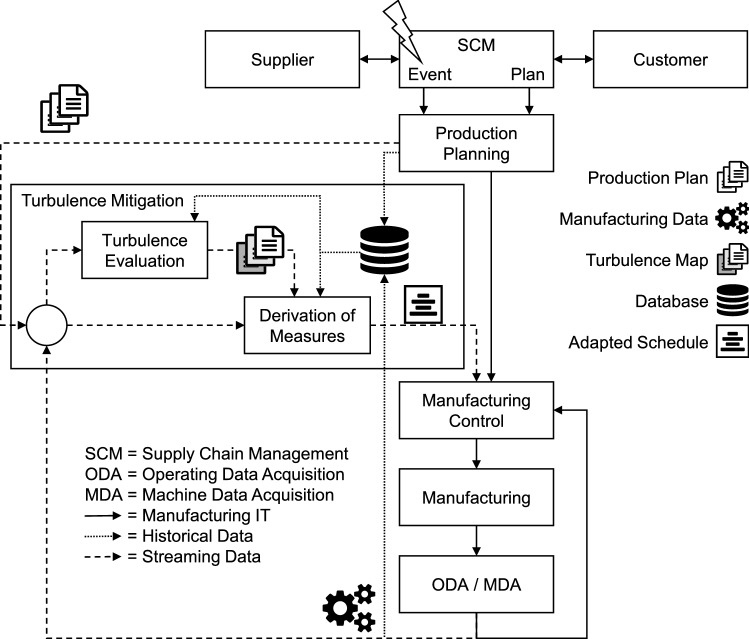
Integration of turbulence evaluation and derivation of measures in production planning and control

The greater the uncertainty, the more important it is for supply networks and within these for manufacturing control to be highly responsive [3, 7]. To optimize the manufacturing system’s performance in case of events causing turbulence, manufacturing control is complemented by a two-step process including turbulence evaluation and derivation of measures (cf. Fig. 3). A first step, the so-called turbulence evaluation, evaluates all future orders regarding their probable cause of turbulence. A second step, the so-called derivation of measures, uses this information to predict measures applied to the orders already being in progress. Both together form the so-called turbulence mitigation. Input data for both steps are historical data of the machine and operating data acquisition (data base), occuring events from the supply network, the planned schedule and the current state of the manufacturing system (all streaming data). To make use of the provided data, machine learning approaches are reasonable. Output of the turbulence evaluation are individual turbulence maps for each future order. Besides the historical data, this is the input for the derivation of measures. Therefore, turbulence maps are considered a pre-aggregated and pre-evaluated data source from the derivation of measure’s point of view.

Based on this data and these two steps, turbulence mitigation derives an adapted schedule aiming at mitigating the consequences caused by turbulences in a manufacturing system. Trained to know the system behavior, turbulence mitigation allows for deciding on measures to effectively and efficiently recover a certain performance of the manufacturing system, ensuring its delivery realibiality.

In the final development stage of the system, turbulence mitigation is the obligatory process step between planning and control. That means that the redundant connection between planning and control, which should be maintained during the implementation phase, becomes obsolete. Turbulence mitigation then becomes an integral part of sequential production planning. Manufacturing control will not be replaced (for the time being), because the job shop-wide control as covered by this approach, needs to be complemented by a fine-granular sequencing and dispatching on machine level. Companies can benefit from this approach by a more intelligent manufacturing control enabling manufacturing to be more robust and responsive, leading to a more resilient manufacturing system. This is achieved by including information about the expected system behavior, in addition to the actual event, in derivation of measures.

### Turbulence evaluation

The approach of this paper is strongly determined by the accessibility of data. To contribute to manufacturing system;s robustness, it is necessary to enable automated or even autonomous systems. The evaluation of turbulences presented here uses historical manufacturing data. After the tool’s initialization, it works without human interference for static systems. Only in case of changes to the strategic goal, and thus to the Key Performance Indicators (KPIs), parameter adjustments are necessary. Other weightings take effect in the calculation of the deviation from relevant indicators. For example, if the main goal shifts from low inventory to a higher degree of service, increasing stock levels would no longer be considered as turbulent as opposed to a lower adherence to schedules.

The customer orders exclusively provide the information base for turbulence evaluation in manufacturing. They represent the volatile load of the manufacturing system and can be processed as time series of variant indices (input data). The manufacturing system’s response can also be described as time series representing states and output of the manufacturing system at any point of time. Within this data set, it is possible to detect functional dependencies between input on the one hand and states and response on the other hand. This represents the simple examination of causes and effects within the regarded manufacturing system. The examination of the functional behavior of the system is mathematically based on correlation and regression studies between the time series and uses tools of signal processing. Consequently, there are three main obstacles to overcome. First, time series do not have mutually equal sampling rates. Second, cause and effect usually have a time delay and third, not all functional dependencies are linear. To adjust the sampling rates, it is possible to use computing power or different alignment functions if some information loss is acceptable. Time delays can be identified by applying convolution operations. Linear dependencies easily can be detected with conventional determination of the correlation coefficients. Non-linear statistical dependencies require other measures like Kendall rank correlation or local regression methods.

**Fig. 4:**
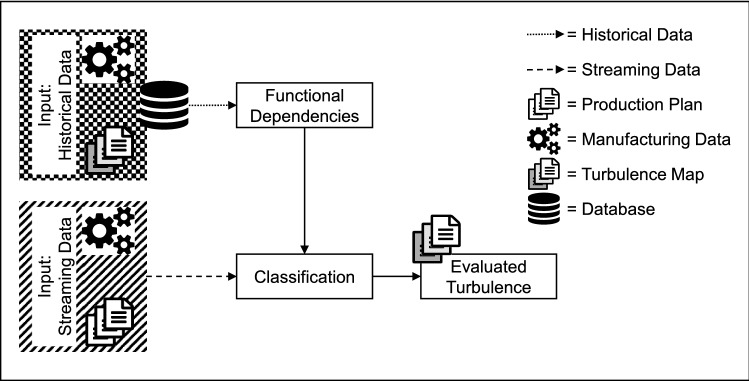
Procedure of turbulence evaluation

The functional dependencies of input data representing the customer orders and their effects on the system’s response are the basis for the classification of orders. It is necessary to define optimum operation points for each goal index and their accepted deviation and weightings. This determines the degree of turbulences: High deviation means a high degree of turbulence. The chosen method for the classification is random forests. The learning data set contains the input values as features and their effects on KPIs as targets. With this dataset, a random set of decision trees (random forest) can be derived. To evaluate the predicted turbulence, the features of a new customer order and the current manufacturing system’s features form the input data of the classifier. The results of the examination of the functional dependencies help to create decision trees of high information gain as only those features are used that have an impact on the goal indices. Figure 4 describes the whole procedure from customer orders as a source of input data to the evaluated turbulences.

It should be considered that this procedure still requires humans in the loop. The system is not designed to define optimum operation points and the degrees of turbulences. Still, it can cope with changes of the system since these changes are represented in the historical data, which is permanently used to update the decision trees. The outcome of the method is a turbulence map for each customer order before its start into the manufacturing system. It can be used as an input variable for planning and control methods.

### Derivation of measures

To derive measures for short-term application, not only an internal control loop between manufacturing control and manufacturing as described in Sect. 2.2 is necessary. Additionally, an adaptive control loop integrating supply network events causing turbulence into manufacturing control is needed. This control loop consists of a controller to derive measures as well as a controlled system consisting of manufacturing control and manufacturing. More precisely, the controller interacts with sequence deviation in manufacturing control. Additionally, operating and machine data acquisition serve as measuring elements [28]. Building on the aptitude described in Sect. 2.3, the learning ability of the controller is based on reinforcement learning. Applied to the derivation of measures, this means that effects can be dealt with that are only visible after orders have been fully processed. Additionally, the system has the ability to learn without labelled data.

**Fig. 5:**
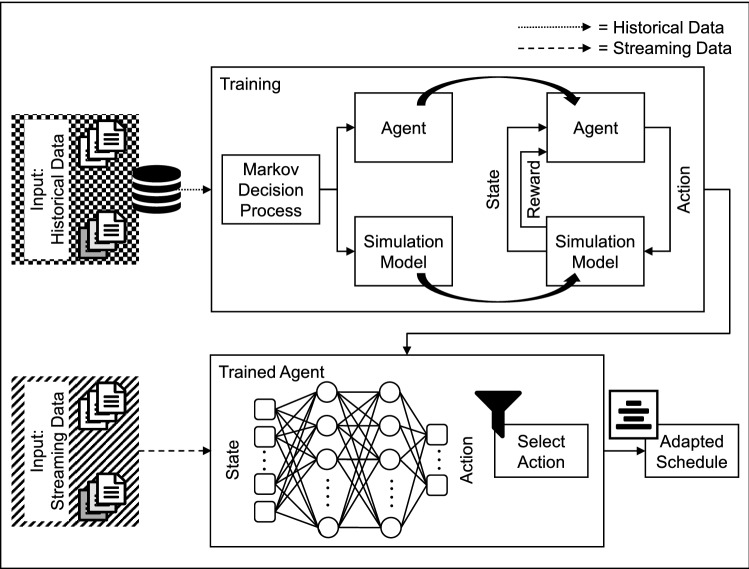
Implementing and applying a reinforcement-learning-based derivation of measures for turbulence mitigation

To implement and apply a reinforcement-learning-based derivation of measures, a training phase as well as an application of the trained agent are necessary (cf. Fig. 5). Although historical data is used to train the agent with a simulation model of the manufacturing system, the trained agent is applied to live streaming data.

As a foundation for reinforcement learning, the MDP has to be designed based on manufacturing data as well as turbulence mapping. The MDP describes states in a state space, actions in an action space and rewards for a learning agent interacting with its environment [43]. Consequently, manufacturing data and turbulence mappings must be found in state space. Actions taken by the agent are leading to a new state including a reward. The outcome of this design is the agent forming the controller as well as a simulation model of the manufacturing system for the agent to interact with in training. This includes an initial set of hyper parameters to use within the agent’s algorithm. To train the agent, it continuously interacts with the simulation model of the manufacturing system to evaluate various actions and their respective rewards. Thereby, the agent is rewarded for minimizing turbulences, meaning to stabilize delivery reliability of the manufacturing system. Training is an iterative process that has to be repeated thousands of times until the reward is maximized. Since training starts without any prior knowledge, the simulation model is necessary. Thus, training can be carried out without negative effects on the manufacturing system. Before transfering the application, the agent must be validated. In addition to statistical validation, the suitability of the agent for the problem at hand must be checked in particular. For this purpose, the simulation model is used to simulate the same scenario with and without intervention by the agent. If the agent performs better, it can be transferred to the application.

Consequently, the controller can be deployed to its application in a real manufacturing environment. Therefore, it no longer interacts with a simulation model. Instead, it uses manufacturing control as an interface to interact with the manufacturing system. Then, actions are performed by executing control methods in manufacturing control, states are constructed by data gathered from machine data acquisition and operating data acquisition within manufacturing control.

## Data requirements

Both the turbulence evaluation and the derivation of measures use two kinds of data: First, historical data of input values and KPIs to teach the system. Second, data about the production orders and current attributes of manufacturing system’s state (near real time, called streaming data). This also includes data on events from the supply network.

**Fig. 6:**
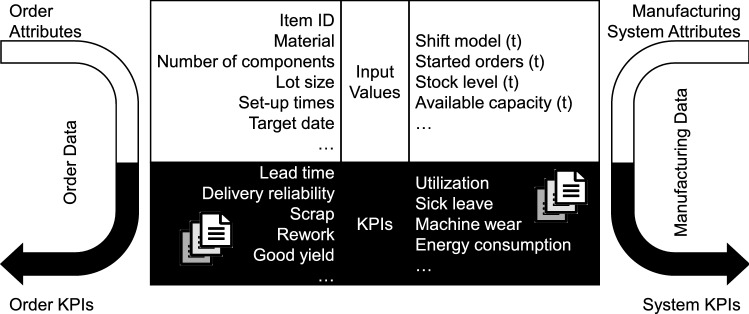
Required data for the approach: order and manufacturing data

Order data and manufacturing data are different (cf. Fig. 6). Order data relates to specific orders (both historical and current). It contains input values describing an unlimited number of order attributes. To gain information from this data, it needs to describe the product, volume of the order and various time attributes. Examples for the product description are the product’s mass, material, color, number of components, lot size. Time attributes could be delivery time or scheduled production finish. Order KPIs describe the order’s attributes after they went through the system. Typical KPIs are lead time, delivery reliability and quality rate.

Manufacturing data is a set of time series. That means that both the manufacturing system’s attributes and the system’s KPIs are states at a specific point in time, which must be assignable. Changes of these states are caused by events. Typical input values of the manufacturing system are inventory, work in process, shift model and available capacities. The output (System KPIs) could be figures like utilization rate, sick rate or energy consumption.

The data needs some preprocessing. The set of training data needs to contain assigned input values and KPIs for both orders and the manufacturing system. This means for each point in time the order attributes and order KPIs as well as the system attributes and KPIs are necessary. These time series need to cover the time in which the orders can be allocated, meaning a minimum time interval of historical data and a minimum frequency to link orders to system’s states. It is important to use data from the time period without significant changes (limited dynamics). Otherwise, the conclusions derived from the data are not valid.

Regarding data handling, turbulence evaluation and derivation of measures work together as follows: Turbulence evaluation assesses the expected turbulence of every order-caused event and provides order KPIs as an input for the derivation of measures. Thus, derivation of measures can utilize the information of production planning and their estimated consequences on lead time, inventory and many more figures. The outcome is an adapted schedule taking into account the turbulences caused by the changes in the customer orders.

## Discussion and benefits

The use case discussed in this paper relates to a manufacturing company including its respective customers and suppliers in a globally distributed supply network. The aim is to mitigate turbulences caused by events from the supply network, such as forecast deviations in delivery dates. Research at Intel, for example, has shown a significant deviation between forecasts and actual demand. Within a time period of ten years they have only matched for 35 min [50]. Therefore, deviations between forecasts and actual demands result in many events that require appropriate responses.

Furthermore, this company’s manufacturing principle can be described as a complex job shop. Job shops are designed for maximum flexibility to deal with the wide product variations encountered [51]. Additionally, complex job shops can be characterized by reentrant order flows, sequence-dependent setup times, different types of processes e.g. single job and batch processing, parallel machines and frequent disturbances [29, 47]. In semiconductor manufacturing, the most common implementation of a complex job shop, these characteristics lead to a cycle time of up to three months [29]. However, with an increasing time horizon, planning accuracy decreases significantly resulting in even more turbulence-causing events [52].

When applying this approach to such a use case, some obstacles need to be mentioned. First, it is highly dependent on data availability and data quality. Consequently, even if the architecture allows for a modular integration into PPC (cf. Sect. 3), this could be prevented by the necessity to acquire data. In a complex job shop, this data depends very much on individual orders and lots. Second, to make this approach work ideally, silo thinking in supply networks has to be broken down and data exchange across companies and manufacturing sites enabled to identify events as soon as possible. However, holistic and continuous information flows are rarely available in industry. The link between supply network and PPC is essentially established by planning objects only.

Once fully implemented, this tool enables a wide range of benefits for manufacturing companies, especially for those organized in complex job shops. First, it allows for an optimization of PPC - which hasn’t been in scope so far—by deriving measures for turbulence mitigation on a control level. Challenges of complex job shops such as reentrant flows and long cycle times, normally interpreted as challenges, can be seen as potential for corrective measures. Second, this self-learning approach using ML technologies allows to perform well on previously unknown data due to its generalization. In this case, the ML technologies cover the classification by decision trees in turbulence evaluation and reinforcement learning in derivation of measures. As mathematical optimization in complex job shop manufacturing reaches its limits, an approach based on ML is ideally suited here. Third, this approach is scalable regarding data variety and data volume. Scalability regarding data variety is enabled by the generalization mentioned above, whereas scalability regarding data volume is given by the architectural design based on the lambda architecture. In semiconductor industry as a typical example of complex job shop manufacturing, the near real-time acquisition of large amounts of data has been standard for many years [29]. Fourth, by using standardized interfaces as proposed e.g. by Bauer et al. [53], a plug-and-play integration with existing PPC IT systems can be achieved.

In sum, this approach with its intelligent manufacturing control allows for a constant adaption of manufacturing to its outer circumstances and, therefore, enables more resilient manufacturing. By maintaining delivery reliability even with the occurence of turbulences, the manufacturing system thus contributes to the resilience of the entire supply network.

## Conclusion and outlook

This work shows an approach to utilize operating and machine data for PPC to improve a manufacturing system’s resilience in a supply network. Starting point is a scheduled order sequence for a manufacturing system, which is interfered by customer- or supplier-induced events. They affect the schedule in different ways. Negative consequences such as deviations of KPIs from their intended values are called turbulences and are to be avoided. This paper deals with the question of how turbulence evaluation can be used to improve the derivation of measures by taking them into account in re-scheduling. The idea is to add a functional module, turbulence mitigation, which combines turbulence evaluation and derivation of measures by using historical and streamed near real-time data from orders and the manufacturing system. The approach suggests decision trees for turbulence evaluation and reinforcement learning for to derive measures. As outlined, the proposed method can be applied as a parallel optimization system to improve existing manufacturing control software, including the potential to fully replace it in the future.

However, research in this area has not yet been finished. First, the approach has to be applied to various use cases to validate the benefits. This lays the foundation for testing and enabling transferability to various industries. Second, as ML-based decision models are mostly non-transparent for humans in their decision-making, it takes approaches of explainable artificial intelligence to make them more comprehensible. Third, this approach can be developed into an eco-system, expandable by additional modules with extended functionality. The PPC tool would work like a modular platform offering interfaces designed to connect existing systems to new data processing modules.

## References

[1] Bauernhansl T (2017) Die Vierte Industrielle Revolution: Der Weg in ein wertschaffendes Produktionsparadigma. In: Vogel-Heuser B, Bauernhansl T, ten Hompel M (eds) Handbuch Industrie 4.0. Springer, Berlin, pp 1–31. 10.1007/978-3-662-53254-6_1

[2] Bauernhansl T, Hörcher G, Bressner M, Röhm M (2018). MANUFUTURE-DE: identification of priority research topics for the sustainable development of european research programmes for the manufacturing industry until 2030.

[3] Lödding H (2013). Handbook of manufacturing control: Fundamentals, description, configuration.

[4] Mack O, Khare A, Mack O, Khare A, Krämer A, Burgartz T (2016). Perspectives on a VUCA world. Managing in a VUCA world.

[5] Ivanov D, Dolgui A (2020). Viability of intertwined supply networks: extending the supply chain resilience angles towards survivability. A position paper motivated by COVID-19 outbreak. Int J Prod Res.

[6] Ponomarov SY, Holcomb MC (2009). Understanding the concept of supply chain resilience. Int J Logist Manag.

[7] Chopra S (2018). Supply chain management: strategy, planning, and operation.

[8] Alkan B, Vera DA, Ahmad M, Ahmad B, Harrison R (2018). Complexity in manufacturing systems and its measures: a literature review. Eur J Ind Eng.

[9] Mattsson S, Gullander P, Davidsson A (2011) Method for measuring production complexity. In: International manufacturing conference IMC 28—manufacturing Sustainability (Dublin)

[10] Seitz KF, Nyhuis P (2015). Cyber-physical production systems combined with logistic models—a learning factory concept for an improved production planning and control. Procedia CIRP.

[11] Gröger C (2015) Advanced Manufacturing Analytics: Datengetriebene Optimierung von Fertigungsprozessen. Stuttgart, Univ., PhD, (Eul, Lohmar, 2015)

[12] Permin E, Bertelsmeier F, Blum M, Bützler J, Haag S, Kuz S, Özdemir D, Stemmler S, Thombansen U, Schmitt R, Brecher C, Schlick C, Abel D, Poprawe R, Loosen P, Schulz W, Schuh G (2016). Self-optimizing production systems. Procedia CIRP.

[13] Cadavid JP Usuga, Lamouri S, Grabot B, Pellerin R, Fortin A (2020). Machine learning applied in production planning and control: a state-of-the-art in the era of industry 4.0. J Intell Manuf.

[14] Brinzer B, Schneider K (2019). Komplexitätsbewertung in der Produktion. ZWF Zeitschrift für wirtschaftlichen Fabrikbetrieb.

[15] Kluth A, Jäger J, Schatz A, Bauernhansl T (2014). Evaluation of complexity management systems—systematical and maturity-based approach. Procedia CIRP.

[16] Ashby WR (1956). An introduction to cybernetics.

[17] Wiendahl HH (2007). Turbulence germs and their impact on planning and control—root causes and solutions for PPC design. CIRP Ann.

[18] Wiendahl HH (2011). Auftragsmanagement der industriellen Produktion: Grundlagen, Konfiguration, Einführung.

[19] Panteli M, Mancarella P (2015). The grid: stronger, bigger, smarter?: presenting a conceptual framework of power system resilience. IEEE Power Energ Mag.

[20] Holling CS (1996) Engineering resilience versus ecological resilience. In: Engineering within ecological constraints

[21] Walker B, Holling CS, Carpenter SR, Kinzig AP (2004). Resilience adaptability and transformability in social-ecological systems. Ecol Soc.

[22] Passos DS, Coelho H, Sarti FM (2018) From resilience to the design of antifragility: the eighth international conference on performance, safety and robustness in complex systems and applications, April 22–26, Athens, Greece

[23] Hedtstück U (2017). Complex event processing: verarbeitung von Ereignismustern in Datenströme.

[24] Ropohl G (2012) Allgemeine Systemtheorie: Einführung in transdisziplinäres Denken. Edition Sigma, Berlin

[25] Schuh G, Lödding H, Stich V, Reuter C, Schmidt O, Potente T, Franzkoch B, Brosze T, Thomas C, Wesch-Potente C, Brecher C, Klocke F (2011). High resolution production management. Wettbewerbsfaktor Produktionstechnik.

[26] Pritschow G, Wiendahl HP (1995). Application of control theory for production logistics—results of a joint project. CIRP Ann Manuf Technol.

[27] Niehues MR, Blum M, Teschemacher U, Reinhart G (2018). Adaptive job shop control based on permanent order sequencing. Prod Eng Res Devel.

[28] Bauer D, Bauernhansl T, Sauer A (2020). Approach for an adaptive control loop between supply network and manufacturing. Procedia CIRP.

[29] Mönch L, Fowler JW, Dauzère-Pérès S, Mason SJ, Rose O (2011). A survey of problems, solution techniques, and future challenges in scheduling semiconductor manufacturing operations. J Sched.

[30] Otto A (2003). Supply chain event management: three perspectives. Int J Logist Manag.

[31] Buckel T (2012) Zum Potential von Event-Driven Architecture für komplexe Unternehmensnetzwerke, in Multikonferenz Wirtschaftsinformatik 2012: Tagungsband der MKWI, ed. by D.C. Mattfeld, S. Robra-Bissantz (Gito, Berlin, 2012). 10.24355/dbbs.084-201301081451-0

[32] Baumgraß A, Botezatu M, Ciccio C Di, Dijkman R, Grefen P, Hewelt M, Mendling J, Meyer A, Pourmirza S, Völzer H (2016) Towards a methodology for the engineering of event-driven process applications. In: Reichert M, Reijers HA (eds) Business process management workshops, lecture notes in business information processing, vol 256. Springer, Cham, pp 501–514. 10.1007/978-3-319-42887-1_40

[33] Konovalenko I, Ludwig A (2019). Event processing in supply chain management—the status quo and research outlook. Comput Ind.

[34] Overbeek S, Janssen M, Tan YH (2012). An event-driven architecture for integrating information, processes and services in a plastic toy supply chain. Int J Cooper Inf Syst.

[35] Linden M, Neuhaus S, Kilimann D, Bley T, Chamoni P (2010) Event-driven business intelligence architecture for real-time process execution in supply chains. In: van der Aalst W, Mylopoulos J, Sadeh NM, Shaw MJ, Szyperski C, Abramowicz W, Tolksdorf R (eds) Business information systems, lecture notes in business information processing, vol 47. Springer, Berlin, pp 280–290. 10.1007/978-3-642-12814-1_24

[36] Russell SJ, Norvig P (2010). Artificial intelligence: a modern approach.

[37] Döbel I, Leis M, Vogelsang MM, Neustroev D, Petzka H, Riemer A, Rüping S, Voss A, Wegele M, Welz J (2018). Maschinelles Lernen: Eine Analyse zu Kompetenzen.

[38] Goodfellow I, Bengio Y, Courville A (2016). Deep learning.

[39] Le Roux N, Bengio Y, Fitzgibbon A, Sra S, Nowozin S, Wright SJ (2012). Improving first and second-order methods by modeling uncertainty. Optimization for machine learning.

[40] Bauer H, Ranade P, Randon S (2012) Big data and the opportunities it creates for semiconductor players, McKinsey on Semiconductors (Autumn 2012), p 46

[41] Oladipupo T (2010) Types of machine learning algorithms (INTECH Open Access Publisher). 10.5772/9385

[42] Criminisi A, Shotton J, Konukoglu E (2011). Decision forests for classication, regression, density estimation, Manifold learning and semi-supervised learning.

[43] Sutton RS, Barto AG (2018). Reinforcement learning: an introduction.

[44] Arulkumaran K, Deisenroth MP, Brundage M, Bharath AA (2017). Deep reinforcement learning: a brief survey. IEEE Signal Process Mag.

[45] Kaelbling LP, Littman ML, Moore AW (1996). Reinforcement learning: a survey. J Artif Intell Res.

[46] Huan SH, Sheoran SK, Wang G (2004). A review and analysis of supply chain operations reference (SCOR) model. Supply Chain Manag.

[47] Mönch L, Fowler JW, Mason SJ (2013). Production planning and control for semiconductor wafer fabrication facilities: modeling, analysis, and systems.

[48] Schenk M, Schürmeyer M, Bauhoff F, Schuh G, Stich V (2012). Koordination interner Produktionsnetzwerke. Produktionsplanung und -steuerung 1.

[49] Bauer D, Maier F, Bauernhansl T, Waschneck B, Ponsignon T, Gürster D, Oberegger B, Felsberger A, Reiner G (2017) Concept and possible application of an automated framework to influence production dispatch based on supply chain events. In: 7th International conference on industrial engineering and systems management (IESM 2017), Saarbrücken, October 11–13 2017, pp 87–92. 10.5281/zenodo.1035179

[50] Wilding R (1998). The supply chain complexity triangle: uncertainty generation in the supply chain. Int J Phys Distrib Logist Manag.

[51] Groover MP (2010). Fundamentals of modern manufacturing: materials, processes, and systems.

[52] Schuh G, Potente T, Thomas C, Hauptvogel A, Prabhu V (2013). Cyber-physical production management. Advances in production management systems. Sustainable production and service supply chains.

[53] Bauer D, Stock D, Bauernhansl T (2017). Movement towards service-orientation and app-orientation in manufacturing IT. Procedia CIRP.

